# Items

**Published:** 1909-10

**Authors:** 


### ITEMS.

MESSRS. BATTLE & CO., Saint Louis, have issued another of their dislocation charts, number 10 of the series. These drawings are in color and may be had on application to the publishers.

---

THE Aspirin patent has been sustained in an elaborate opinion by the Honorable A. L. Sanborn, judge of the United States Circuit Court of the Northern district of Illinois. The action is entitled Farbenfabriken of Elberfeld Company, *complainant, vs.* Edward A. Kuehmsted, *defendant.*

The decision confirms the rights granted to the complainant under the patent to exclusively sell acetyl salicylic acid under this name or any other names whatsoever.

The rights of Farbenfabriken of Elberfeld Company are equally infringed, if the unlicensed product is sold under the name of acetyl salicylic acid or under complainants trade-marked name of aspirin.

The decision is of importance to the drug trade as well as to physicians and should be read by all concerned. It can be had on application to Messrs. Farbenfabriken, of Elberfeld Company, 117 Hudson Street, New York.

